# Using a novel bio-based cationic flocculant for food industry wastewater treatment

**DOI:** 10.1038/s41598-024-69558-2

**Published:** 2024-08-21

**Authors:** Ghada E. Ahmed, Gamal K. Hassan, Elshimaa H. Gomaa, Samar A. Aly, Sanaa Y. Salem, Entsar E. Badr, Karim M. Aboelghait, Ahmed A. fify

**Affiliations:** 1 Canal Higher Institute for Engineering and Technology, Suez, Egypt; 2 Water Pollution Research Department, National Research Centre, Cairo, 12622 Egypt; 3 Department of Chemistry, Faculty of Science for Girls, Al-Azhar University, Cairo, 11754 Egypt; 4 Genetic Engineering and Biotechnology Research Institute, University of Sadat City, El Sadat, Egypt

**Keywords:** Wastewater treatment, Bio-based cationic surfactant, Organic contaminants, Chemical treatment, Sedimentation, Environmental sciences, Environmental social sciences

## Abstract

Wastewater from the food industry is considered harmful to human health and aquatic life, as well as polluting water and soil. This research is centered around finding an affordable and easy physicochemical method for dealing with waste generated by the food industry. To accomplish this goal, a new bio-based flocculant called 4-benzyl-4-(2-oleamidoethylamino-2-oxoethyl) morpholin-4-ium chloride was created using sustainable sources, specifically crude olive pomace oil. Its chemical structure was confirmed using various spectroscopic techniques such as FTIR, ^1^H-NMR, mass spectra, and ^13^C-NMR. This new bio-based cationic flocculant was combined with alum to act as a coagulant in the waste treatment process. Also, a study was conducted to determine the optimal conditions for the coagulation-flocculation process parameters, namely, pH and alum dosage, on COD and removal efficiency. The results showed that the optimal conditions for flocculation were achieved at pH 5.8, with 680 mg/L alum and 10 mg/L of commercial flocculant dose compared to only 5 mg/L of a new bio-based cationic flocculant. A comparison was made between the new bio-cationic flocculant and a commercial CTAB one for treating wastewater in the food industry. The study found that the new bio-based cationic flocculant was more effective in reducing the chemical oxygen demand, achieving a reduction of 61.3% compared to 54.6% for using a commercial cationic flocculant. Furthermore, using a new bio-based cationic flocculant costs only 0.49 $/g, which is less than the present cationic flocculant, which costs 0.93 $/g. The adoption of this new flocculant provides a sustainable alternative to existing industrial wastewater treatment processes

## Introduction

Rapid population growth and industrialization have put pressure on natural resources and imposed enormous challenges on the ecosystem. Global water resources are under great pressure, and one of the growing concerns is the discharge of polluted water into wastewater or water bodies, so combating water pollution is essential^1–3^.

Due to the varying raw materials and production processes used in different foods, wastewater from the food industry often has a complex composition and unpleasant odor^4–6^. Its composition includes a large number of food-suspended solids, various chemical forms of cooking oil, fat, protein, and other organic substances. In recent years, the food industry has experienced a rapid increase in wastewater production. Although the food industry wastewater is low in toxicity, it usually has a high concentration of organic matter (such as sugar, protein, nitrogen, and phosphorus compounds, etc.)^7^, so if such wastewater is directly discharged without treatment, it will bring great challenges to the environment and human health. It can cause eutrophication in water bodies, leading to an anoxic environment and the death of aquatic organisms^8^. Once wastewater-suspended matter settles in the river, it decomposes anaerobically, leading to odor and quality deterioration^9^. If this wastewater is used to irrigate farmland, it can damage crop growth and groundwater circulation^10^.

Therefore, the selection of appropriate technologies for the treatment of food industry wastewater has become a research hotspot in the field of food, including physical and chemical systems such as coagulation, filtration, evaporation, centrifugation^11^, adsorption^12^, Fenton oxidation^13^, ozone^14^, microalgae cultivation^15^, constructed wetlands^16^, UV disinfection^17^ and microbial fuel cells (MFCs)^18^. However, their use has been restricted due to their high operating costs and the secondary pollutants they produce^19^. Developing economical, efficient, and environmentally friendly wastewater treatment systems for the food industry is becoming more important to address this problem.

In physical treatment techniques, the chemicals are removed through the application of physical barriers and naturally occurring forces including gravity electrical attraction, and van der Waal forces. The chemical structure of the target compounds is generally unaffected by the mechanisms underlying physical treatment. Certain situations result in a change in the physical state, such as vaporization, and others frequently induce dispersed substances to clump together, like filtration. Sedimentation, flotation, membranes and adsorption are examples of physical wastewater treatment techniques as well^20,21^.

The olive oil industry is a vital part of agriculture in the Mediterranean region, responsible for more than 90% of the world's olive oil production^22,23^. Crude olive pomace oil is a by-product of this industry, and researchers have studied its physical, chemical, and rheological properties^24,25^. This liquid contains high levels of polyphenols and tannins, has a low pH, and produces a high chemical and biochemical oxygen demand, all of which make it difficult to biodegrade and challenging to manage as wastewater and sewage. The findings from these studies have provided valuable information on the potential uses of crude olive pomace oil in various applications^26^. Recently, some biopolymers can used to achieve sustainable treatment of wastewater and these biopolymers includes, particularly polysaccharides such as starch, cellulose, and chitosan^27^. Some of previous studies have prepared and used bio- flocculants as a promising alternative for other traditional materials. A Nanofibrillated cationic cellulose derivatives was used for domestic wastewater treatment and it was sufficient to reduce dissolved organic carbon by 65%^28^. Moreover, COD was reduced by 47% by using cationic lignin polymers in another study^29^. This trend pave the way for researchers to use a novel bio-flocculant to treat industrial wastewater.

Depending on the authors' knowledge, there is a research void regarding the identification of a low-cost and adaptable system capable of treating food industry wastewater with emphasis on its cost analysis. Therefore, the objectives of this study were to: (i) Propose a novel approach to convert crude olive pomace oil (COPO) into a bio-based cationic flocculant and then evaluate the performance of bio-based cationic flocculants as a new flocculant for the food industry wastewater treatment, and (ii) Examine the potential for achieving better removal efficiency by using bio-based cationic flocculants and alum at a lower dosage in less time. (iii) Fourier Transform Infrared (FTIR), 1H-NMR, and 13C-NMR were analyzed to characterize 4-benzyl-4-(2-oleamidoethylamino-2-oxoethyl) morpholin-4-ium chloride (BOMC) and to elucidate flocculation mechanisms. (iv) Moreover, the study was enriched with an economic study to compare the marketing of the commercial teaser novel with its commercial marketing.

## Materials and methods

### Source of the crude olive pomace oil (COPO)

Crude olive pomace oil (purchased from a factory located in Ismailia Governorate) is a type of oil that is extracted from the residual pomace, which consists of the solid remnants of olives after the initial pressing for extra virgin olive oil, with solvent (like hexane).

### Synthesis of bio-based cationic flocculant

**Scheme 1:**
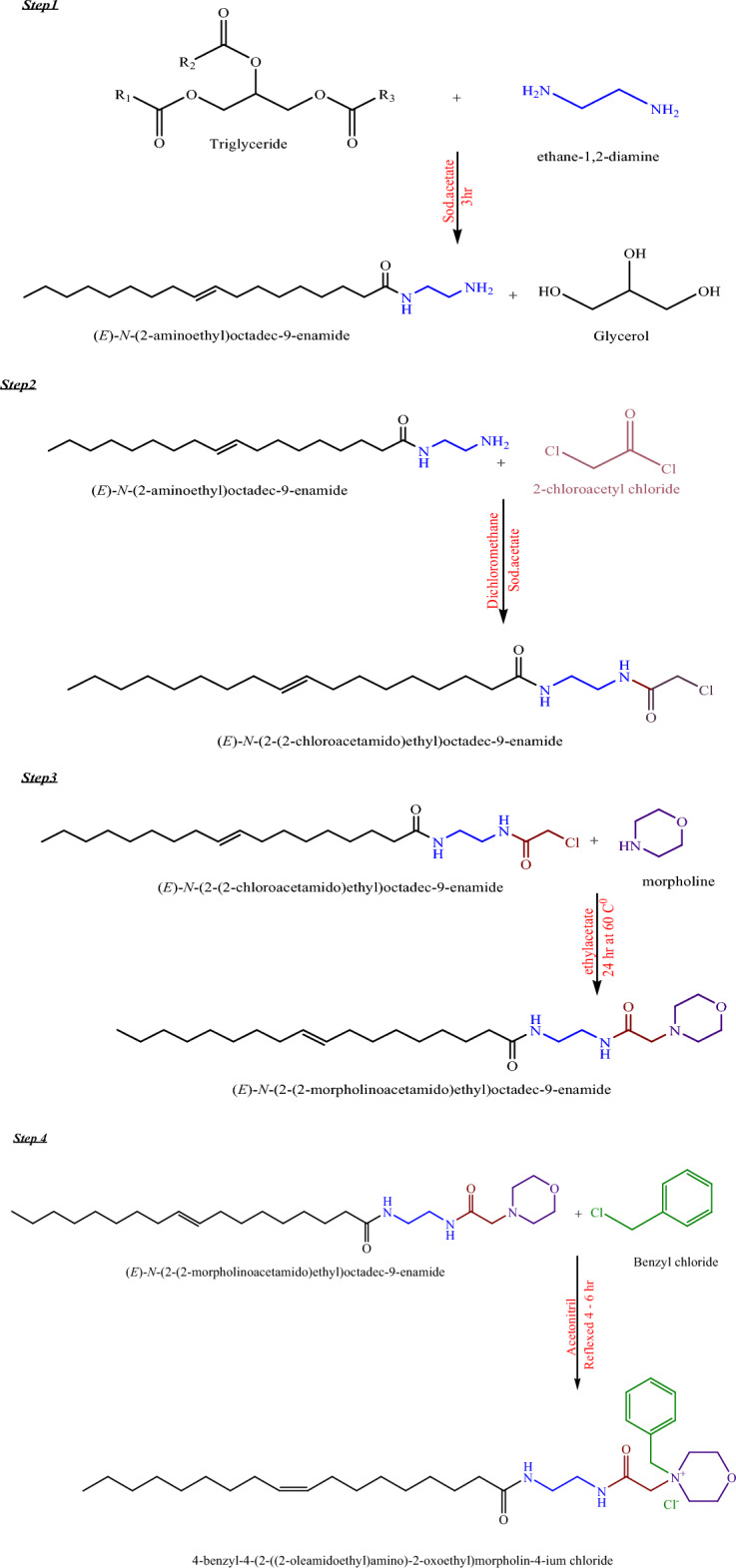
Synthesis of bio-based cationic flocculant.

The synthesis of bio-based cationic flocculant was illustrated in Scheme 1. The cationic flocculant was prepared in the following four steps:

#### Amidation of olive pomace oil

The olive pomace oil (0.01 mol) was added to a neck round-bottom flask along with ethylene diamine (0.31 mol) and anhydrous sodium acetate (0.2 wt.%). The mixture was then stirred at 160 °C for 3 h. Once the reaction was complete, the resulting product was dissolved in an appropriate amount of dichloromethane. This solution was then added to a separation funnel and washed with water to remove any impurities. Finally, the solvent was removed under reduced pressure to yield the desired product, AEA^30^.

#### Synthesis of N-(2-(2-chloroacetamido) ethyl) octadic-9-enamide (CAEO)

AEA (3.783 mmol) was dissolved in an appropriate amount of dichloromethane, while K_2_CO_3_ (5.675 mmol) was dissolved in water. The resulting aqueous solution was added to the organic solution, resulting in a two-phase mixture. The mixture was cooled to a temperature of 5 °C. Next, a solution of chloroacetyl chloride (5.675 mmol) in an appropriate amount of dichloromethane was added slowly dropwise to the cooled solution over approximately 30 min while keeping the temperature at 5 °C. After completing the addition, the reaction mixture was left to stir at room temperature for approximately 2 h.

The aqueous solution was then separated from the mixture and washed with dichloromethane twice using 25 mL of dichloromethane each time. All the organic solutions were then combined and washed with water twice, using 50 mL of water each time. After the washing process, the mixture was passed over anhydrous Na_2_SO_4_ to remove any remaining traces of water. The solvent was then removed under reduced pressure to obtain the desired product, CAEO^31^.

#### Synthesis of (E)-N-(2-(2-(morpholin-1-yl) acetamido) ethyl) octadec-9-enamide:(MAEO)

Morpholine (4.356 g, 0.05 mol) was reacted with CAEO (0.02 mol) in an appropriate amount of ethyl acetate under reflux for 24 h. After completion of the reaction, the crude mixture was cooled to room temperature. The solvent was then removed under reduced pressure using a rotary flash evaporator. The crude mixture was then washed twice with 20 mL of deionized water, and then by 10 mL of aqueous methanol (1:1 water: methanol). Subsequently, the mixture was dissolved in chloroform and dried using Na_2_SO_4_. Finally, the solvent was removed under reduced pressure at 60 °C in a rotary flash evaporator to obtain the desired product, MAEO^32^.

#### Synthesis of 4-benzyl-4-(2-oleamidoethylamino-2-oxoethyl) morpholin-4-ium chloride (BOMC)

Benzyl chloride (10 mmol) and MAEO (10 mmol) were dissolved in an appropriate amount of acetonitrile. The reaction mixture was then refluxed for 4–6 h. After the completion of the reaction. After the reaction, the crude residue obtained was mixed with diethyl ether and allowed to stand for some time. The upper diethyl ether layer was then removed, leaving behind a viscous liquid. This liquid was dissolved in acetonitrile and then dispersed into diethyl ether. The trituration-decant procedure was carried out twice more to ensure the complete removal of any residual starting materials. The resulting viscous liquid was then dried under vacuum to obtain the desired product, BOMC^33^.

### Evaluation method of bio-based cationic flocculant

#### Chemical treatment

**Figure 1:**
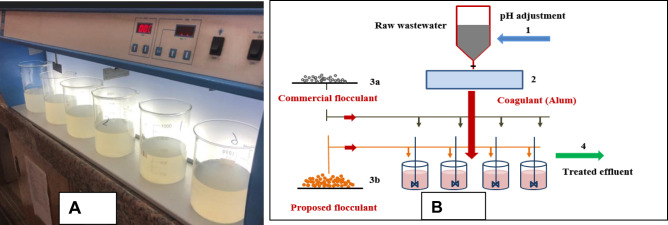
(**A**) Real photo of the entire experimental procedures, showing coagulation/flocculation jar test and (**B**) Schematic diagram of the used system for coagulation/flocculation process.

Chemical treatment, as shown in Fig. 1, is applied by using aluminum sulfate as a coagulant and both commercial surfactant (CTAB) and prepared one (BOMC) as flocculants. The solution pH is adjusted by adding HCl (10%) and/or NaOH (10%). All used chemicals are of analytical grade. The coagulant was combined with raw wastewater at 200 rpm for average 4 min as a flash, followed by slow stirring at 30–40 rpm for 30 min to form flocks. Consequently, 200, 400, 600, 800, and 1000 mg/L of alum were used to investigate the optimum dose. The commercial flocculants was added with the optimum dose from Alum. CTAB was added with a concentration of 1, 2, 5, 10, and 15 mg/L. CTAB was replaced by the prepared material BOMC as a flocculant and for comparison, 1, 2, 5, 10, and 15 mg/L. from the prepared material were used. The mixtures were allowed to settle for 30 min. Turbidity, COD, and TSS were measured to indicate the efficiency of each flocculant.

### Analytical methods

#### Fatty acid composition of crude olive pomace oil (COPO)

Table 1 reports the physicochemical properties of the extracted olive pomace oil, including its density, color, viscosity, saponification value, free fatty acid, iodine value, peroxide value, and fatty acid composition. The fatty acid composition of extracted olive pomace oil was identified using a modified method^34^ with GC model 7890B from Agilent Technologies.

**Table 1:** Composition and characteristics of crude olive pomace oil.

Characteristics	Values
Density (g/cm^3^)	0.912
Color	Greenish-yellow
Viscosity (cP at 25 ℃)	84–97
Composition (% fatty acids)	C_16:0_: 18.52; C1_6:1_: 1.38; C_18:0_: 3.45; C_18:1_: 65.90; C_18:2_: 8.55
Peroxide value	5 milliequivalents of active oxygen/kg oil
Appearance at 20 °C	Limpid
Saponification value	188 mg KOH/g oil
Iodine value (Wijs)	93
Free fatty acids (expressed as oleic acid)	0.33 g/100 g oil

#### Wastewater analysis

A composite sample was collected from a company for industrial food production in the industrial zone at 6th of October city that located in west of Cairo city. The samples were transported to the lab in ice-box for analysis, however, pH was measured on site and the rest of the parameters were measured in the lab. The collected wastewater samples have been analyzed according to APHA^35^.

### Consent to participate

All of the authors consented to participate in the drafting of this manuscript.

## Results and discussion

### Physiochemical analysis of olive pomace oil

Table 1 summarizes the chemical composition and characteristics of olive pomace oil. The oil had a clear, yellowish-green color and no suspended particulates. The COPO displayed a saponification value (SV) of 188. mg of KOH/g of oil and free fatty acid (FFA) content of 0.33 g/100 g, The COPO had an iodine value of 93 g//100 g, indicating a rather high degree of unsaturation. Additionally, the COPO had a low peroxide value of 5 milliequivalents of active oxygen/kg oil, indicating the presence of small amounts of hydro-peroxides that are produced due to oil oxidation during the extraction and storage of the oil. A significant amount of saturated fatty acids, namely stearic acid (3.45%) and palmitic acid (18.52%), were present in the COPO. Conversely, notable levels of unsaturated fatty acids, including oleic acid (65.90%), linoleic acid (8.55%), and palmitoleic acid (1.38%), were present in the COPO.

### Chemical structure confirmation of synthesized bio-based cationic flocculant

The synthesis of a bio-based flocculant can be achieved in four steps. First, the recovered oil undergoes amidation with ethylenediamine which is done by a nucleophilic substitution reaction in which the alkoxy group of alcohol is replaced by NH_2_ of ethylenediamine leading to the formation of 1-(2-aminoethylamino) octadec-9-en-2-one (AEA). Next, the AEA is condensed with chloroacetylchloride via an electrophilic substitution reaction where the protons of the amino group of AEA are replaced by the chloroacetyl group to produce N-(2-(2-chloroacetamido) ethyl) octadec-9-enamide (CAEO). Afterward, the CAEO is reacted with morpholine via nucleophilic substitution reaction to replace chloride with the nucleophile (nitrogen atom of morpholine), yielding N-(2-(2-(morpholin-1-yl) acetamido) ethyl) octadec-9-enamide (MAEO). To quaternarize the tertiary nitrogen, benzyl chloride is added to the MAEO mixture, resulting in the formation of 4-benzyl-4-(2-oleamidoethylamino-2-oxoethyl) morpholin-4-ium chloride (BOMC). The chemical reaction is illustrated in Scheme (1), and the chemical structures of the prepared compounds are confirmed using FT-IR and ^1^H-NMR, ^13^C-NMR, and Mass spectra analyses.

#### Chemical structure of 1-(2-aminoethylamino) octadec-9-en-2-one (AEA)

**Figure 2:**
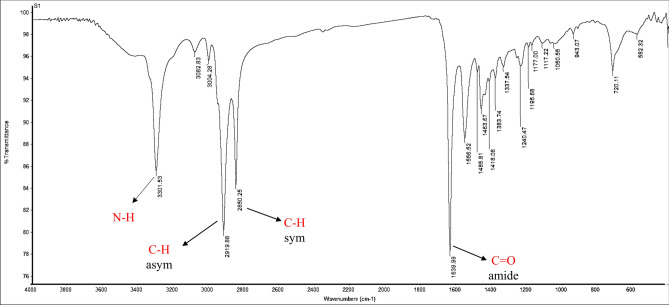
IR spectrum of 1-(2-aminoethyl amino) octadic-9-en-2-one (AEA).

FT-IR (KBr, ν_max_ cm^-1^) Fig. 2: 3301.53 (ν_NH_ stretch), 2919.88, 2850.25 (ν_C–H_ asym. and sym. stretch), 1639.99 (ν_C=O_, amide).

**Figure 3:**
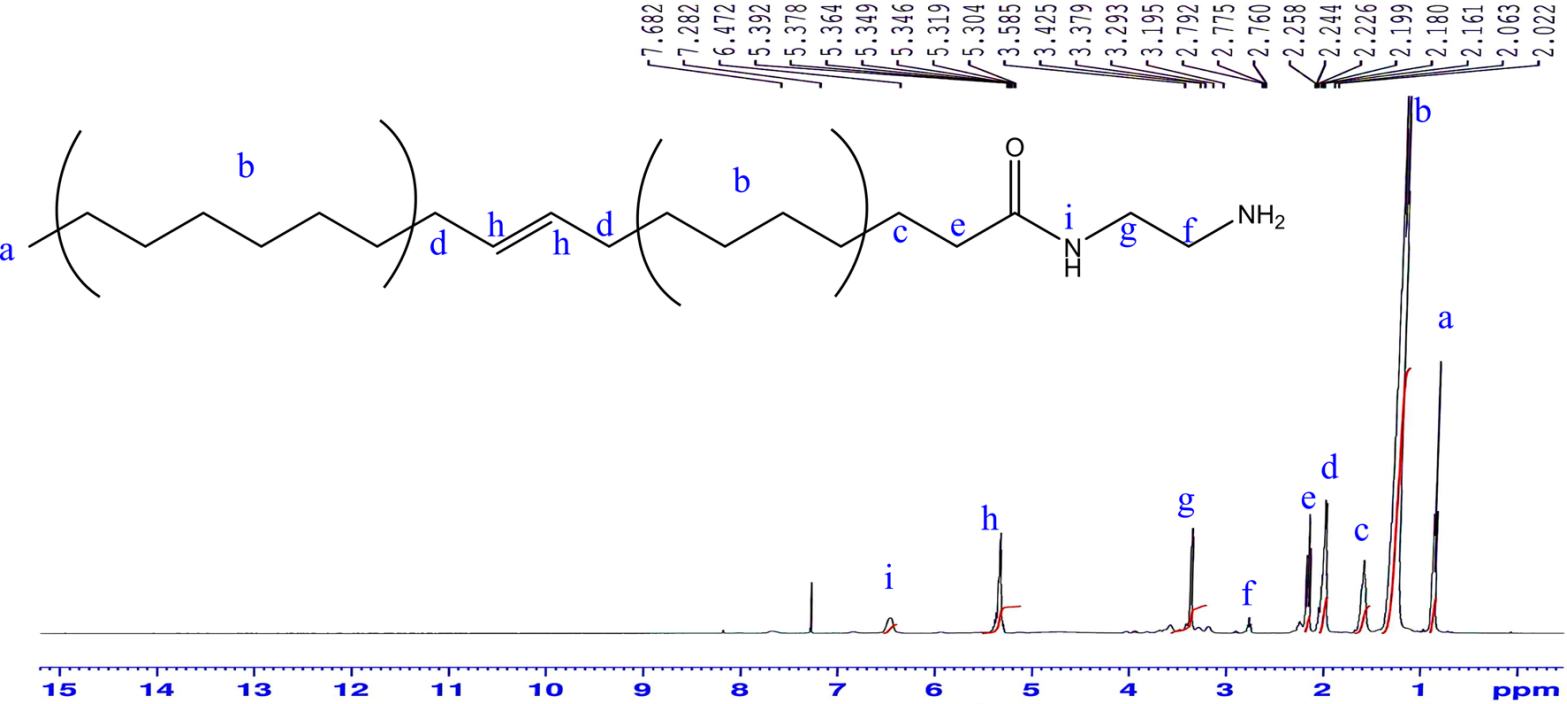
^1^H-NMR spectrum of1-(2-aminoethyl amino) octadic-9-en-2-one (AEA).

^1^H-NMR (CDCl_3_) δ (ppm) Fig. 3: 0.89 (3H; –***CH***_***3***_), 1.27 (20H; –***CH***_***2***_–), 1.61 (2H; –***CH***_***2***_CH_2_CO–), 1.99 (4H; –***CH***_***2***_CH=CH–***CH***_***2***_–), 2.24 (2H; –***CH***_***2***_CO–), 2.79 (2H; NH_2_***CH***_***2***_–CH_2_–), 3.29 (2H; –***CH***_***2***_NH–CO–), 5.36 (2H; –CH_2_ ***CH***=***CH***–CH_2_–), 6.47 (1H; –CH_2_***NH***–CO–).

**Figure 4:**
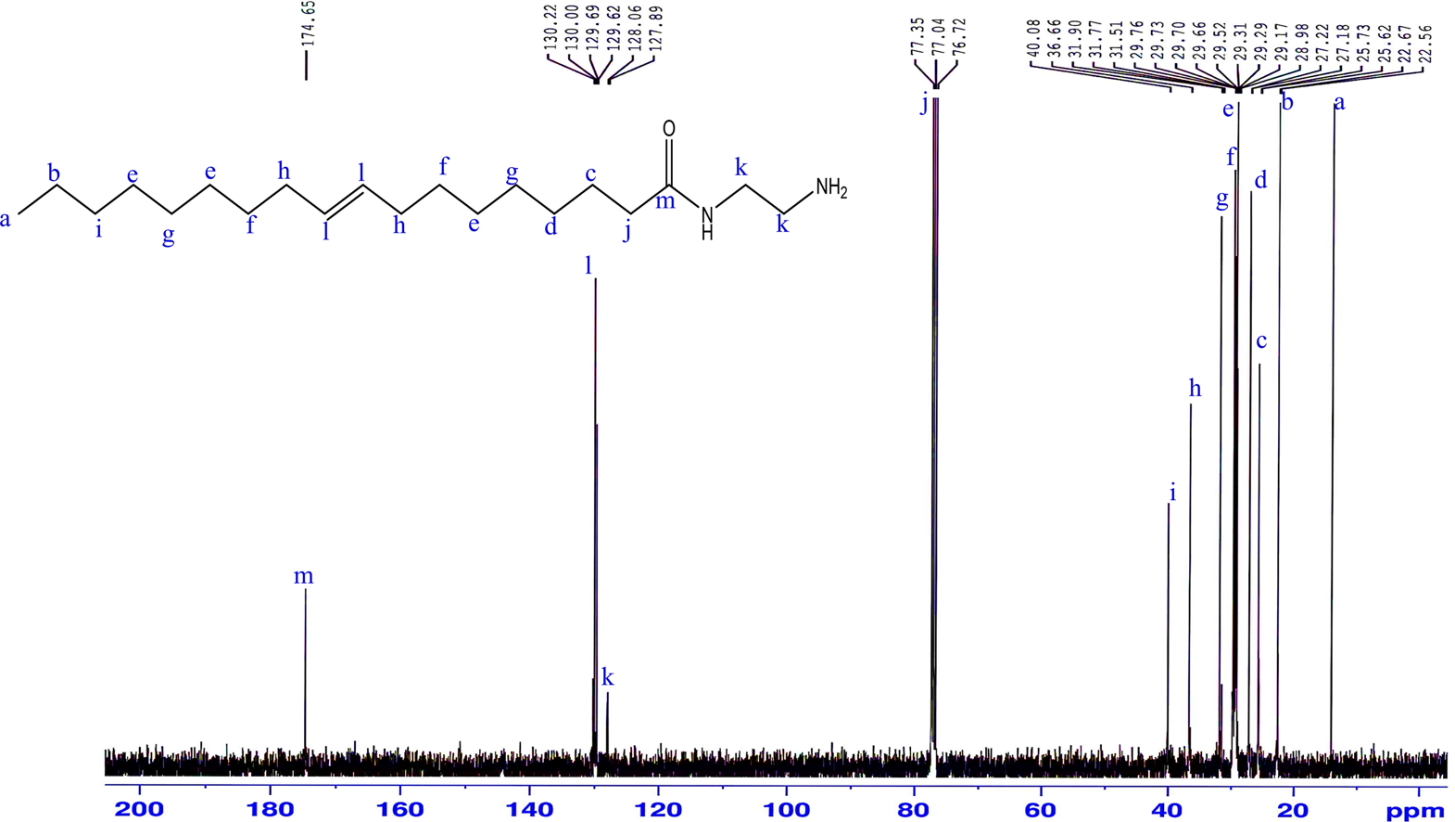
^13^C-NMR spectrum of1-(2-aminoethyl amino) octadic-9-en-2-one (AEA).

**Figure 5:**
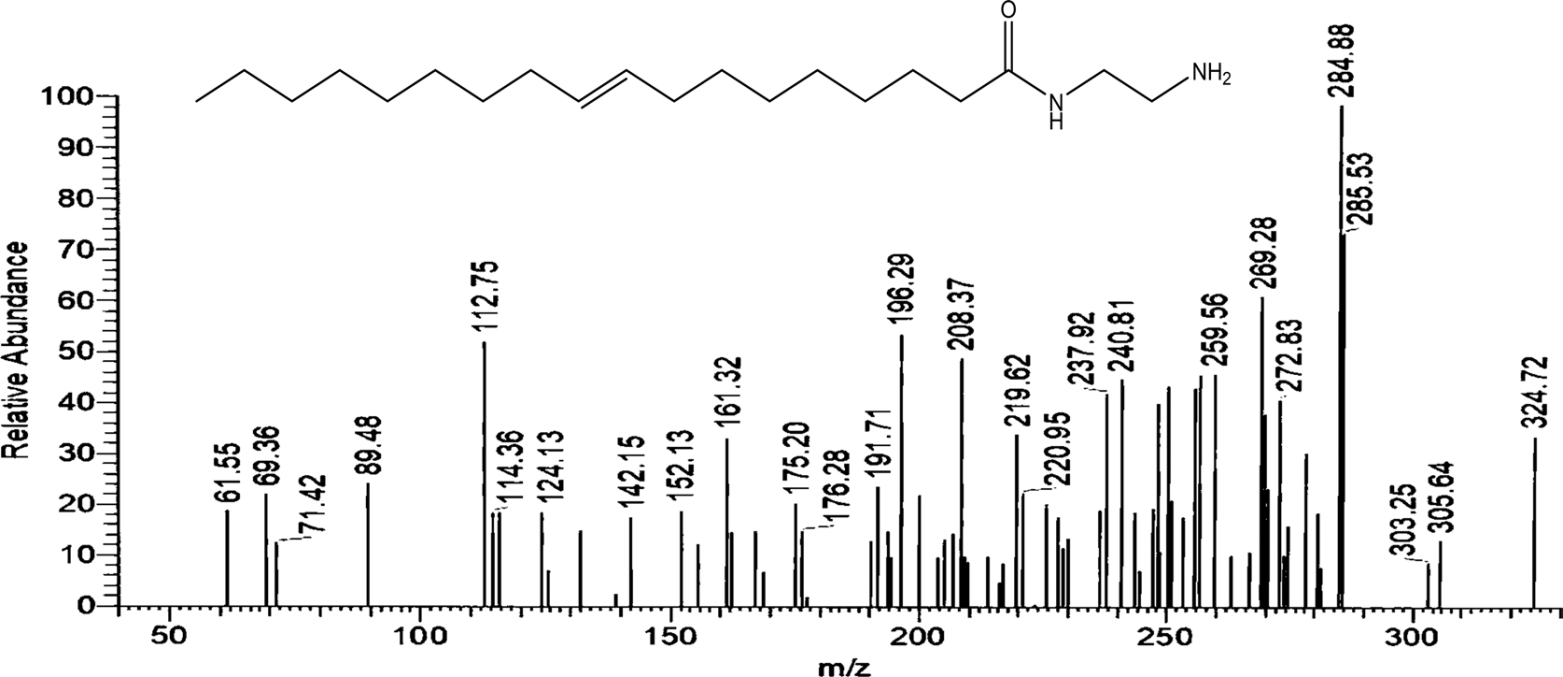
Mass spectrum of 1-(2-aminoethyl amino) octadic-9-en-2-one (AEA).

^13^C-NMR (CDCl_3_) δ (ppm) Fig. 4: 14.11, 22.67, 25.73, 27.22, 29.29, 29.52, 29.76, 29.99, 31.90, 36.66, 40.08, 130.22, 174.65;

The mass spectrum Fig. 5 of ***AEA*** exhibited a molecular ion peak [M^+^] at m/z 324.72 (34.3% C_20_H_40_N_2_O) with a base peak at 284.88 (C_17_H_36_N_2_O),

#### Chemical structure of N-(2-(2-chloroacetamido) ethyl) octadec-9-enamide (CAEO)

**Figure 6:**
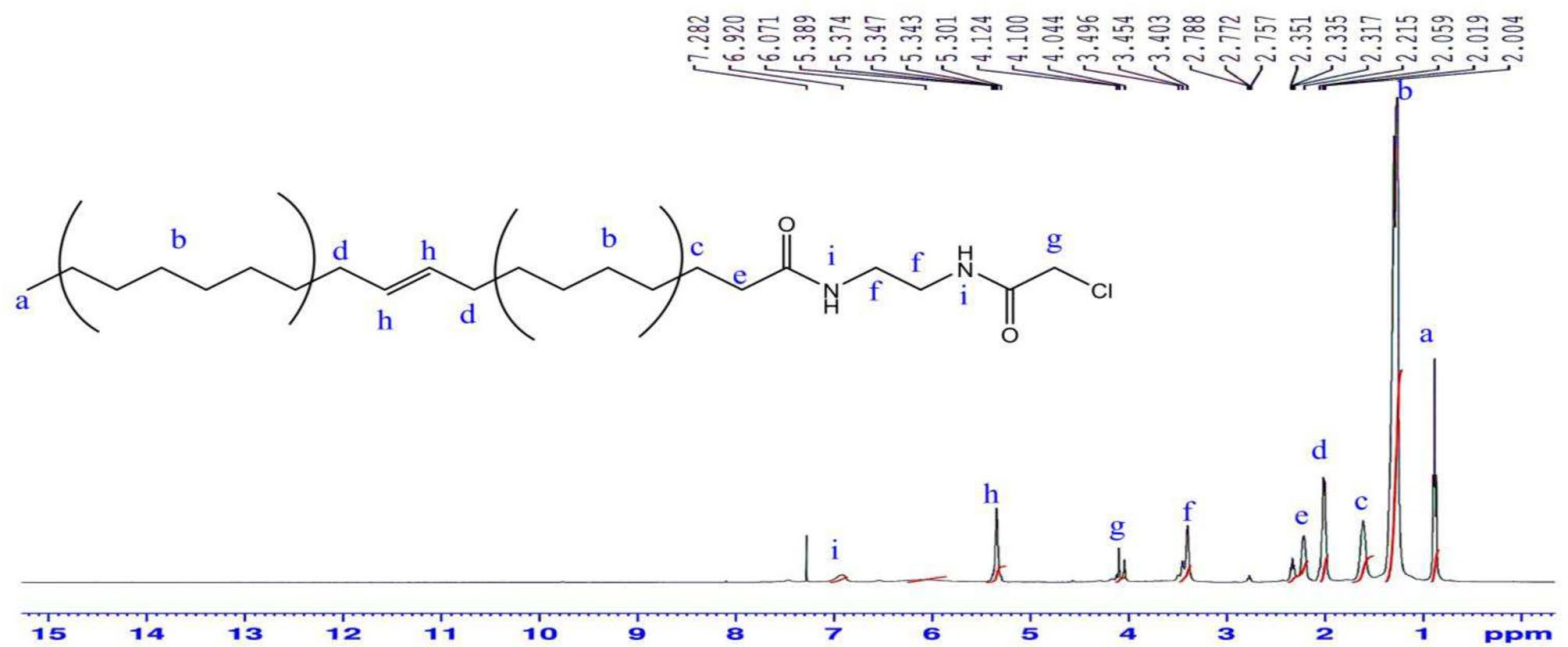
IR spectrum of N-(2-(2-chloroacetamido) ethyl) octadic-9-enamide (CAEO).

FT-IR (KBr, ν_max_ cm^−1^) Fig. 6: 3299.29 (ν_NH_ stretch), 2920.72, 2851.05 (ν_C–H_ asym. and sym. stretch), 1639.34 (ν_C=O_, amide).

**Figure 7:**
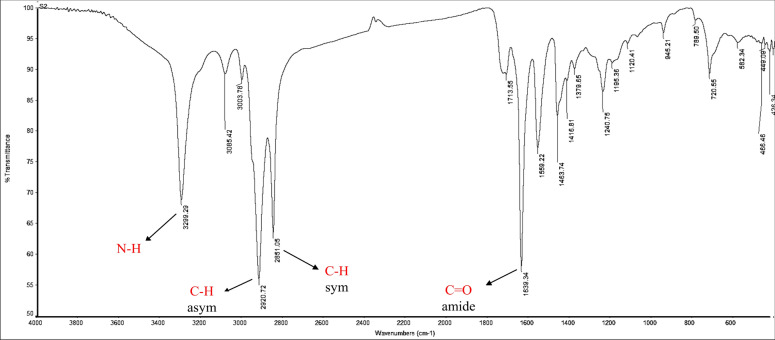
^1^ H-NMR spectrum of N-(2-(2-chloroacetamido) ethyl) octadic-9-enamide (CAEO).

**Figure 8:**
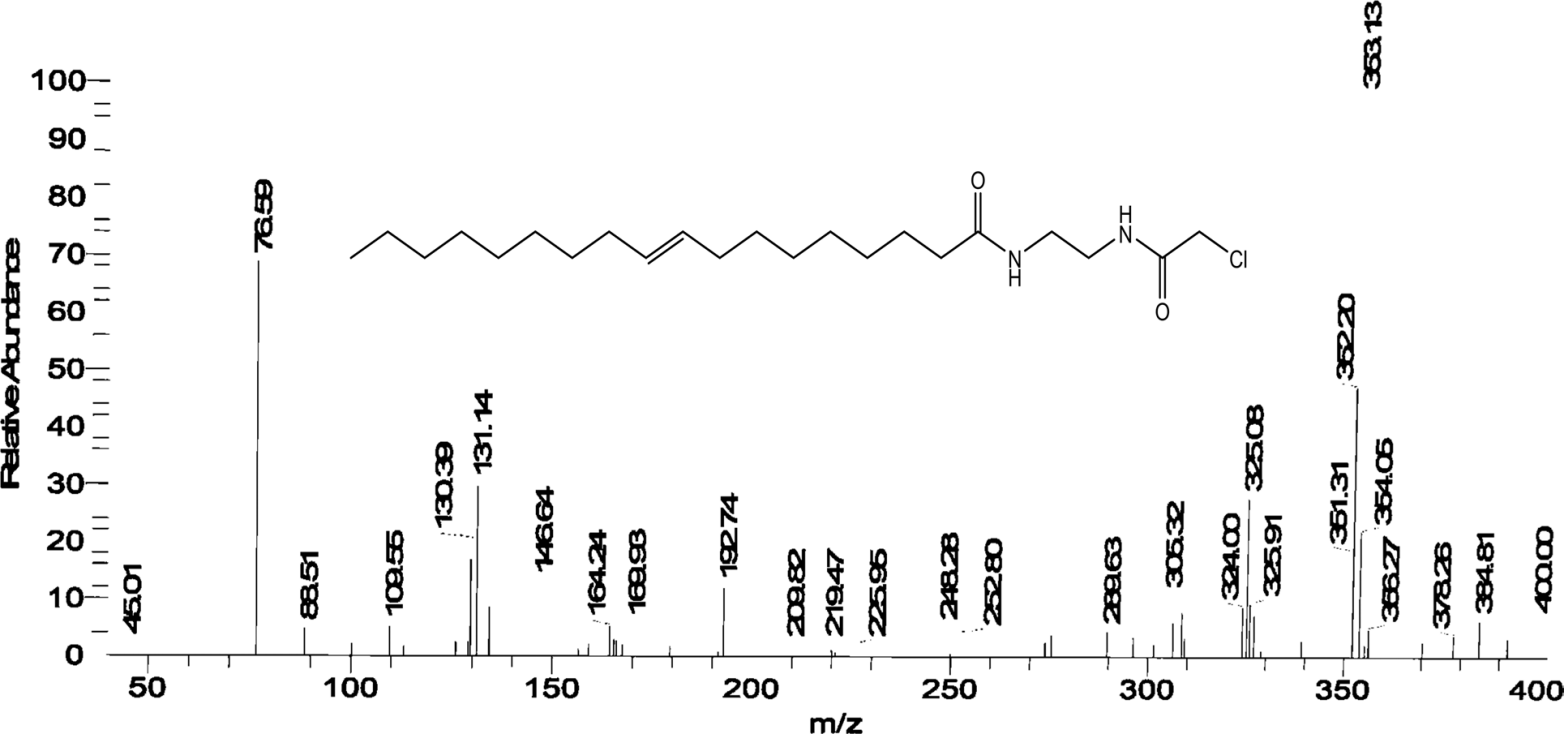
Mass spectrum of N-(2-(2-chloroacetamido) ethyl) octadic-9-enamide (CAEO).

^1^H-NMR (CDCl_3_) δ (ppm) Fig. 7: 0.87 (3H; –***CH***_***3***_), 1.27 (20H; –***CH***_***2***_–), 1.61 (2H; –***CH***_***2***_CH_2_CO–), 2.01 (4H; –***CH***_***2***_CH=CH–***CH***_***2***_–), 2.33 (2H; –***CH***_***2***_CO–), 3.45 (4H; NH***CH***_***2***_–**CH**_**2**_–NH), 4.1 (2H; Cl–***CH***_***2***_–CO–), 5.34 (2H; –CH_2_ ***CH***=***CH***–CH_2_–), 6.9 (2H; CO–***NH***–CH_2_–CH_2_***NH***–CO–) The mass spectrum Fig. 8 of ***CAEO*** exhibited a molecular ion peak [M^+^] at m/z 400 (6.79% C_22_H_41_N_2_O_2_Cl) with a base peak at 353.13 (C_19_H_30_N_2_O_2_Cl).

#### Chemical structure of N-(2-(2-(morpholin-1-yl) acetamido) ethyl) octadec-9 enamide:(MAEO)

**Figure 9:**
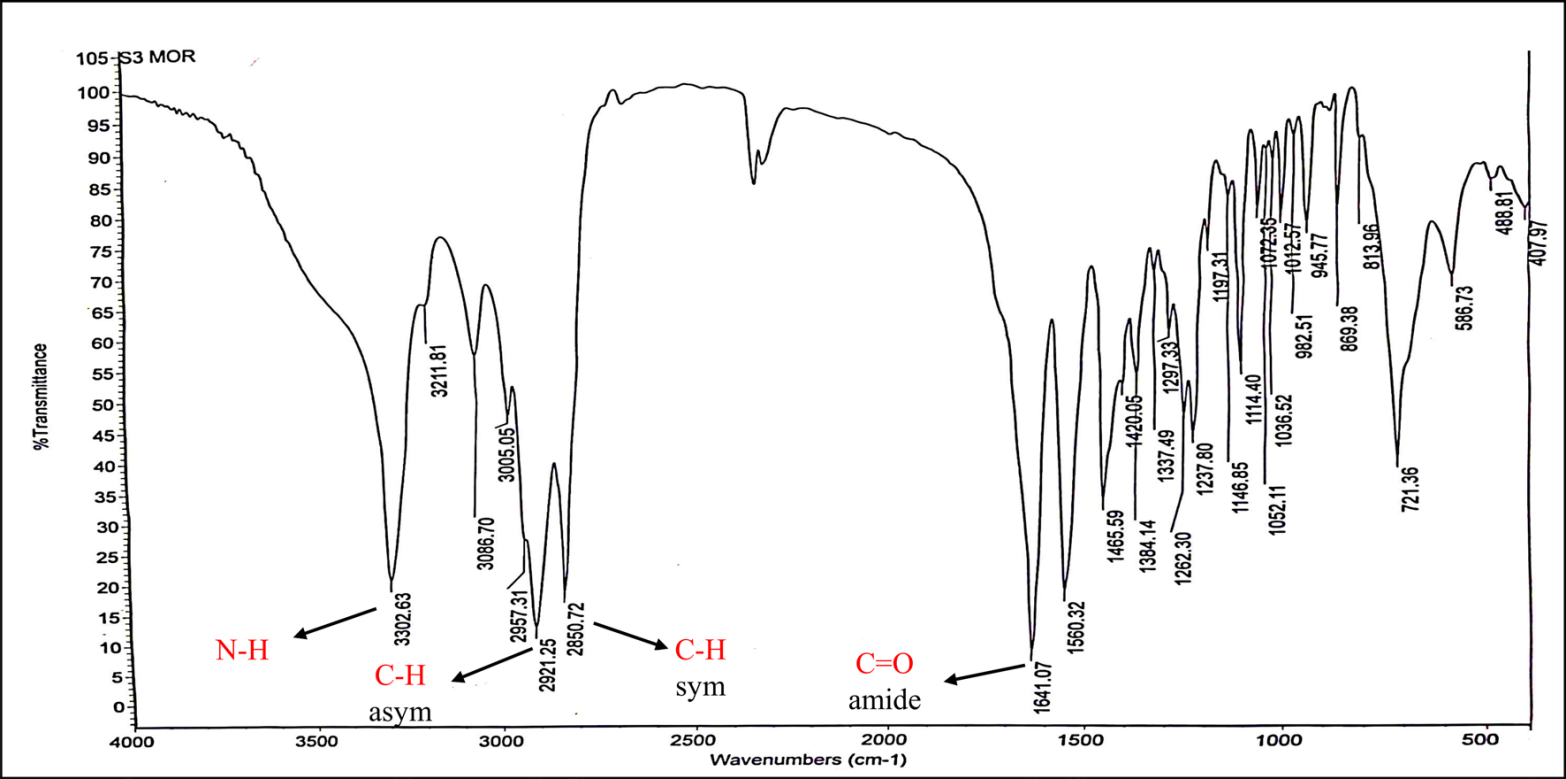
IR spectrum of N-(2-(2-(morpholine-1-yl) acetamido) ethyl) octadic-9 enamide: (MAEO).

FT-IR (KBr, ν_max_ cm^-1^) Fig. 9: 3302.63 (ν_NH_ stretch), 2921.25, 2850.71 (ν_C–H_ asym. and sym. stretch), 1641.07 (ν_C=O_, amide).

**Figure 10:**
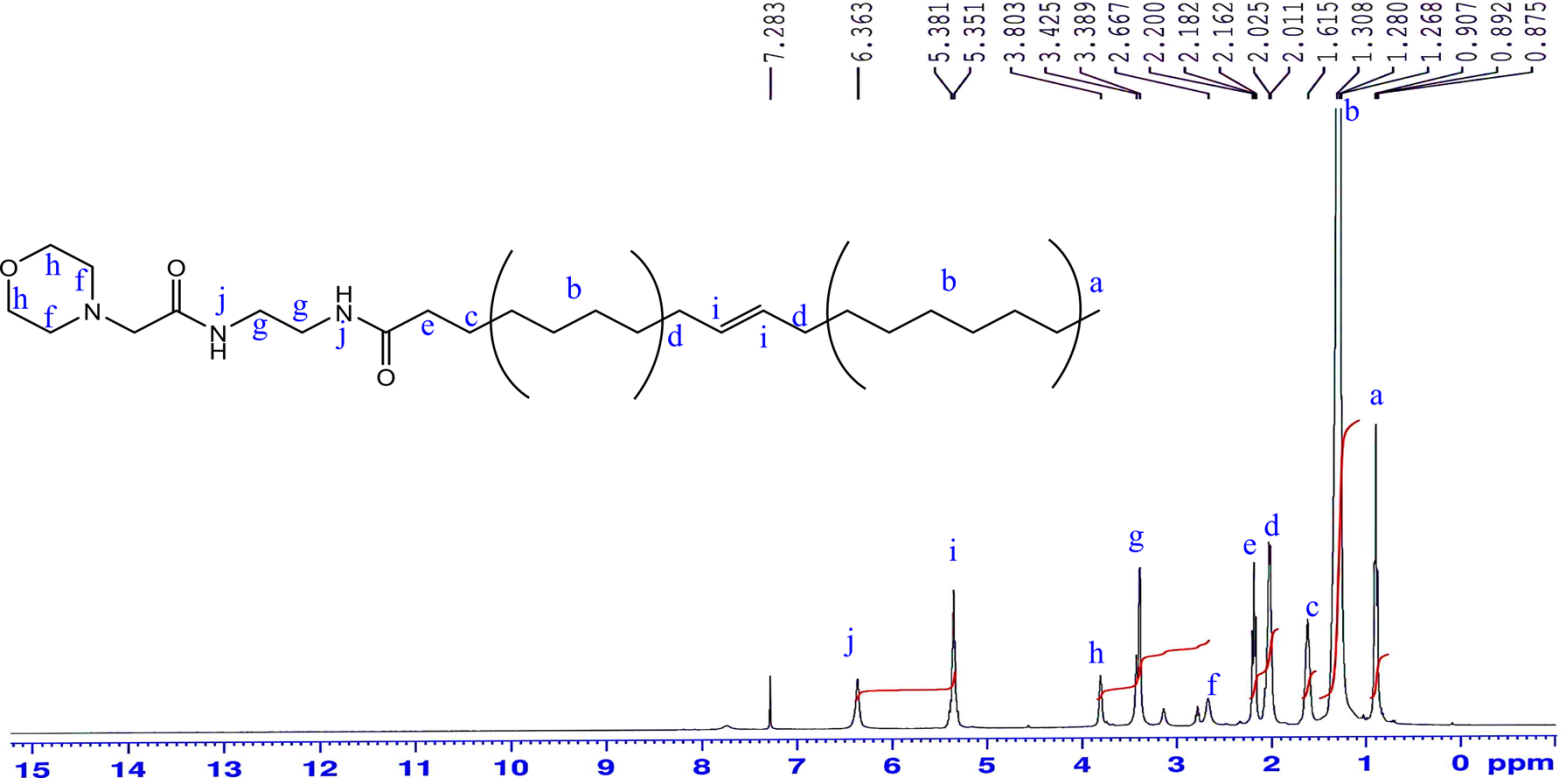
^1^H-NMR spectrum of N-(2-(2-(morpholine-1-yl) acetamido) ethyl) octadic-9 enamide: (MAEO).

^1^H-NMR (CDCl_3_) δ (ppm) Fig. 10: 0.89 (3H; –***CH***_***3***_), 1.28 (20H; –***CH***_***2***_–), 1.61 (2H; –***CH***_***2***_CH_2_CO–), 2.02 (4H; –***CH***_***2***_CH=CH–***CH***_***2***_–), 2.2 (2H; –***CH***_***2***_CO–), 2.6 (4H; –***CH***_***2***_–N–***CH***_***2***_–), 3.38 (6H; NH***CH***_***2***_–**CH**_**2**_–NH–CO–***CH***_***2***_), 3.8 (4H; ***CH***_***2***_–O–***CH***_***2***_), 5.35 (2H; –CH_2_ ***CH***=***CH***–CH_2_–), 6.36 (2H; CO–***NH***–CH_2_–CH_2_***NH***–CO–).

#### Chemical structure of 4-benzyl-4-(2-oleamidoethylamino-2-oxoethyl) morpholin-4-ium chloride (BOMC)

**Figure 11:**
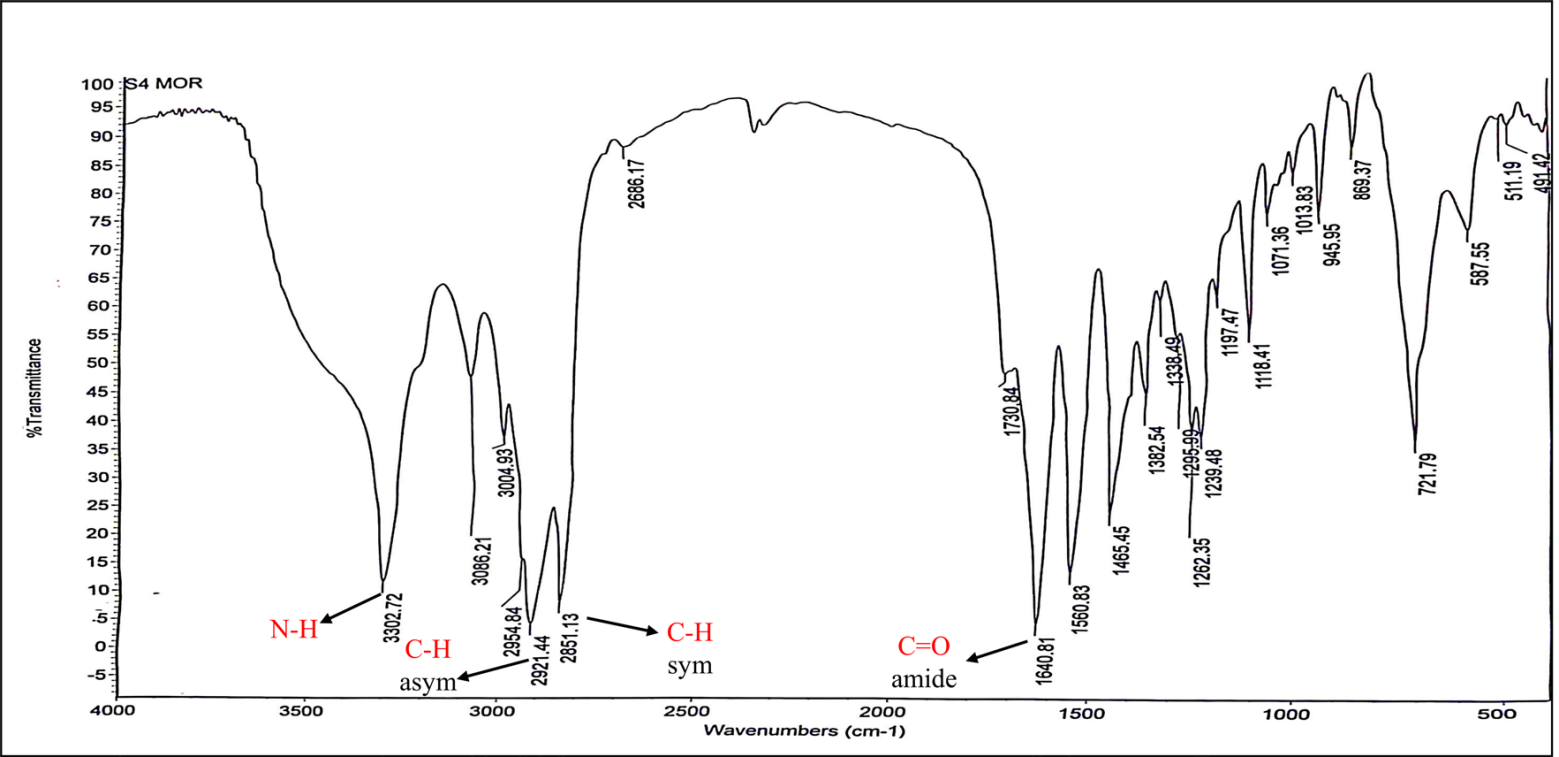
IR spectrum of4-benzyl-4-(2-oleamidoethylamino-2-oxoethyl) morpholin-4-ium chloride (BOMC).

FT-IR (KBr, ν_max_ cm^−1^) Fig. 11: 3302.72 (ν_NH_ stretch), 2921.84, 2851.13 (ν_C–H_ asym. and sym. stretch), 1640.81 (ν_C=O_, amide).

**Figure 12:**
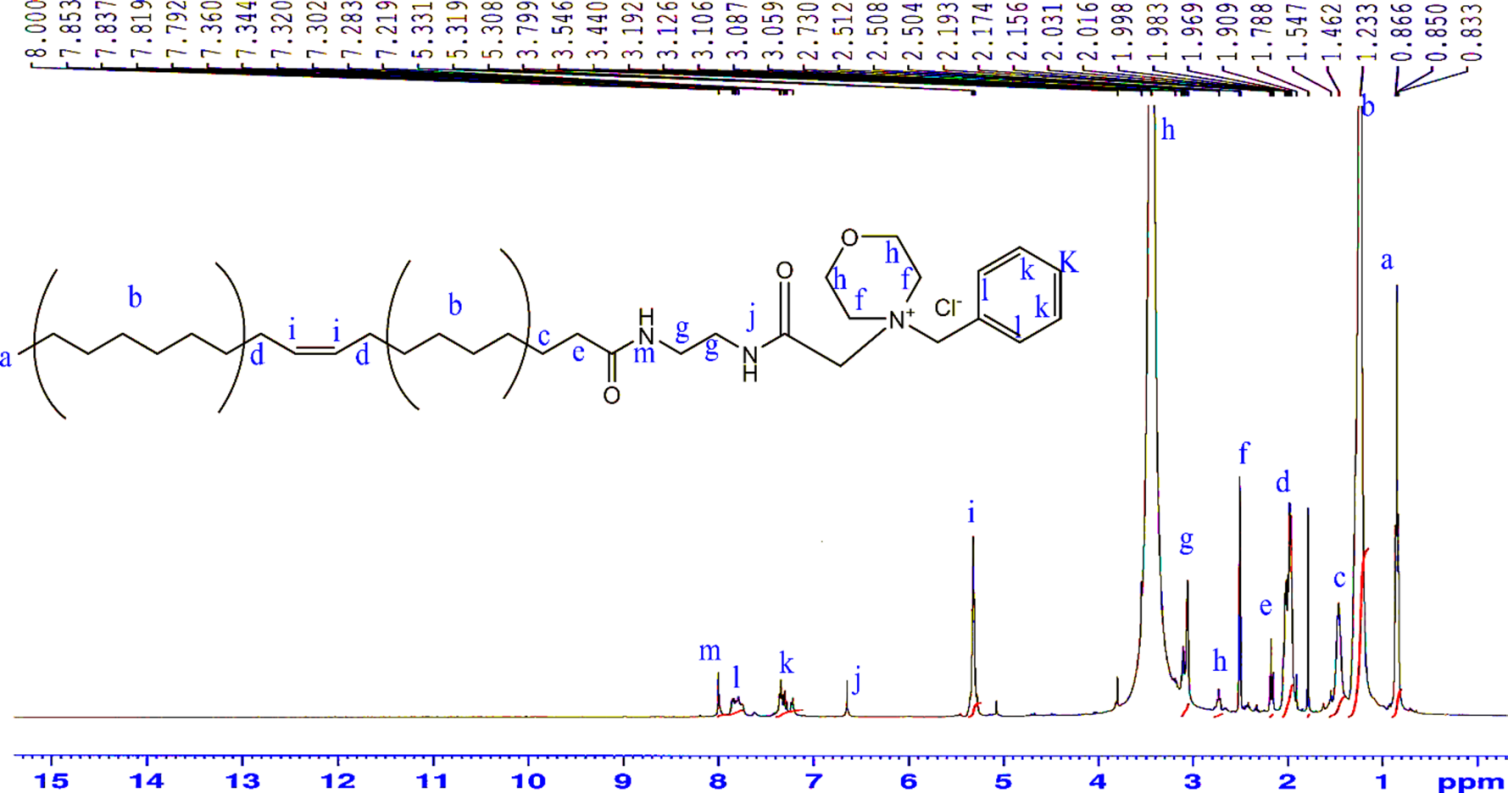
^1^H-NMR spectrum of4-benzyl-4-(2-oleamidoethylamino-2-oxoethyl) morpholin-4-ium chloride (BOMC).

^1^H-NMR (CDCl_3_) δ (ppm) Fig. 12: 0.86 (3H; –***CH***_***3***_–), 1.23 (20H; –***CH***_***2***_–), 1.54 (2H; –***CH***_***2***_CH_2_CO–), 1.92 (4H; –***CH***_***2***_ CH=CH–***CH***_***2***_–), 2.17 (2H; –***CH***_***2***_CO–), 2.5 (4H; –***CH***_***2***_–N–***CH***_***2***_–), 3.12 (4H; NH***CH***_***2***_–***CH***_***2***_–NH), 3.54 (4H; –***CH***_***2***_O***CH***_***2***_–), 3.8 (4H; –***CH***_***2***_N***CH***_***2***_–), 5.3 (m; 2H; CH_2_–***CH***=***CH***–CH_2_), 6.65 (1H; CO–***NH***–CH_2_), , 7.40 (m; 2H; Ar.), 7.60 (m; 2H; Ar.), 8 (1H; –***NH***–CO–CH_2_)

**Figure 13:**
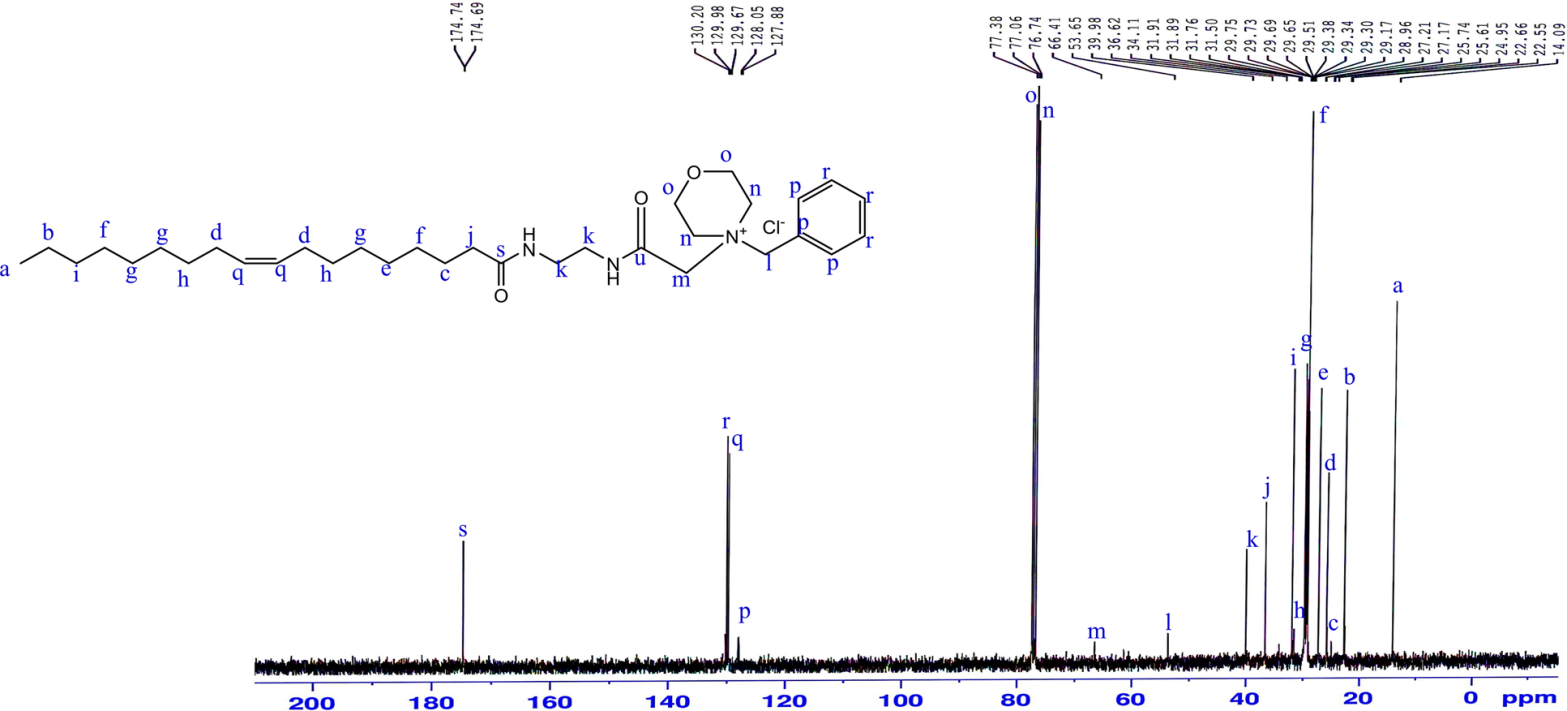
^13^C-NMR spectrum of 4-benzyl-4-(2-oleamidoethylamino-2-oxoethyl) morpholin-4-ium chloride (BOMC).

^13^C-NMR (CDCl_3_) δ (ppm) Fig. 13: 14.09, 22.66, 25.74, 27.20, 29.17, 29.34, 29.51, 29.69, 29.75, 31.89, 34.11, 36.62, 39.98, 53.65, 66.41, 76.74, 77.38, 127.88, 129.67, 130.20, 174.74.

### Treatment of food industry wastewater by coagulation-flocculation process

#### Coagulation-flocculation process using alum with commercial cationic flocculant (CTAB)

**Figure 14:**
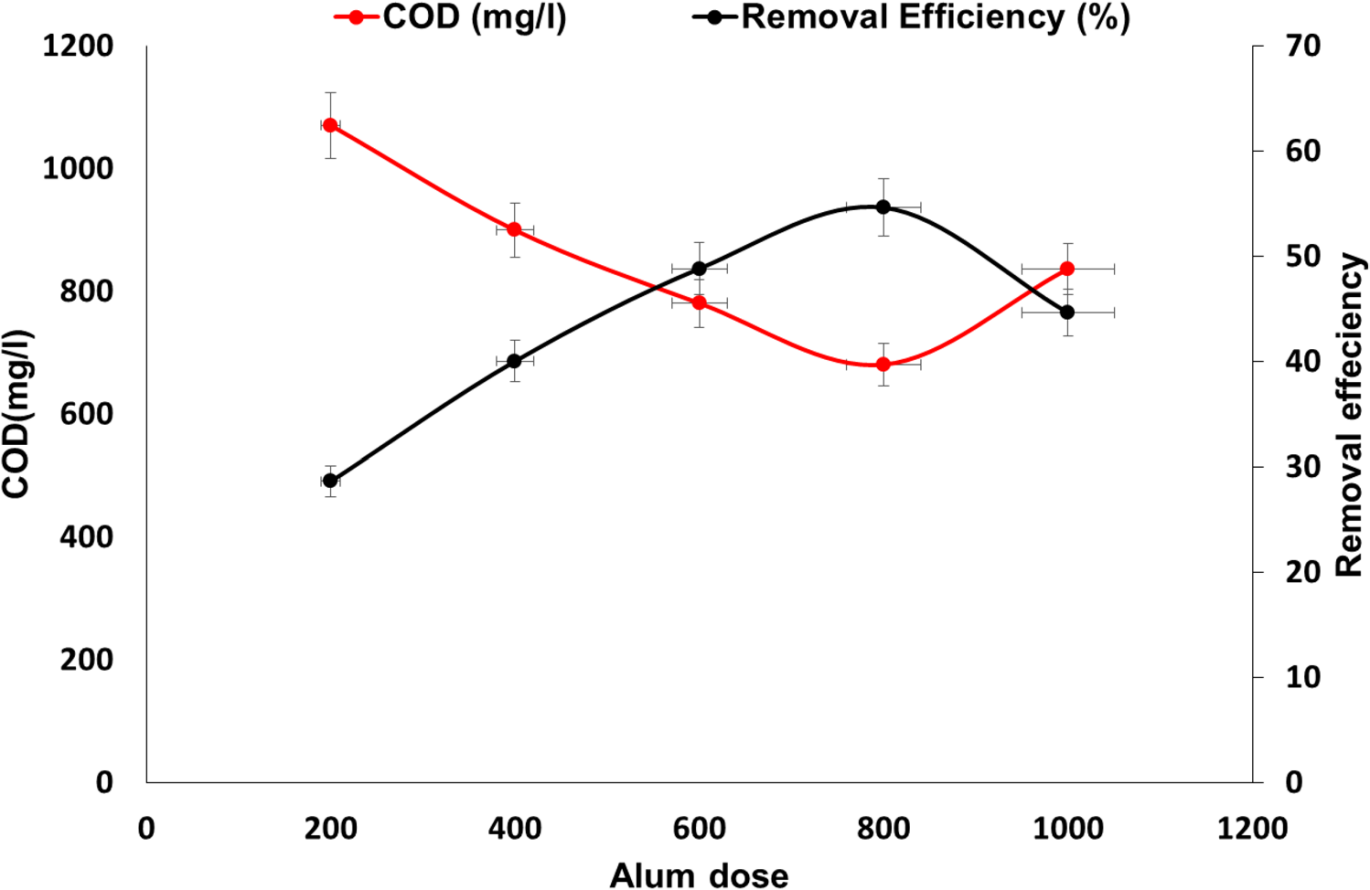
Effect of alum dose (used alone) on COD removal at conditions of (pH 5–5.5, Initial COD 1500 mg/L).

As shown in Fig. 14 and Table 2, many alum doses have been used to optimize the operating conditions, starting from 200 mg/L to 1 g/L. COD concentrations were reduced from 1500 mg/L to 1070, 899, 780, 680, and 836 mg/L using concentrations of alum of 200, 400, 600, 800, and 1000 mg/L respectively. These results show that the best results for the removal of hazardous materials from food industry wastewater were obtained using alum with a concentration of 800 mg/L. The produced pH ranges from 5.0 to 5.8, however, the optimum pH was 5.4 at the concentration of 800 mg/L, and this was the same observation after analysis of TDS produced from the treatment process as shown in Table 1. The reduction of the turbidity was not significant as it was reduced from 266 NTU to ranges from 180 to 190 NTU. These results were comparable with El‑Ezaby et al.^36^ who used the alum for a chemical–coagulation process to treat wastewater from a fruit juice factory as food industry wastewater (and reported that the removal of COD was 57%, however, the removal of COD in the current study reached 54.6%. This confirms that the removal pathway for the same wastewater was the same trend as in other studies, and the same removal was followed by the addition of a flocculant to improve the treatment process.

**Table 2:** Treatment of food industry wastewater using Alum without any flocculant.

Parameter	Raw wastewater	Alum dose (mg/L)				
		200	400	600	800	1000
pH	6.7 ± 1.0	5.8 ± 0.9	5.7 ± 0.9	5.4 ± 0.8	5.2 ± 0.7	5.0 ± 0.5
NTU	266 ± 12	184 ± 8	186 ± 6	180 ± 8	180 ± 8	190 ± 10
TDS	770 ± 15	1060 ± 18	1040 ± 18	1080 ± 20	1110 ± 22	1140 ± 26
TSS	516 ± 24	370 ± 22	310 ± 18	290 ± 28	270 ± 28	315 ± 32
COD	1500 ± 124	1070 ± 105	899 ± 88	780 ± 78	680 ± 66	836 ± 90

**Figure 15:**
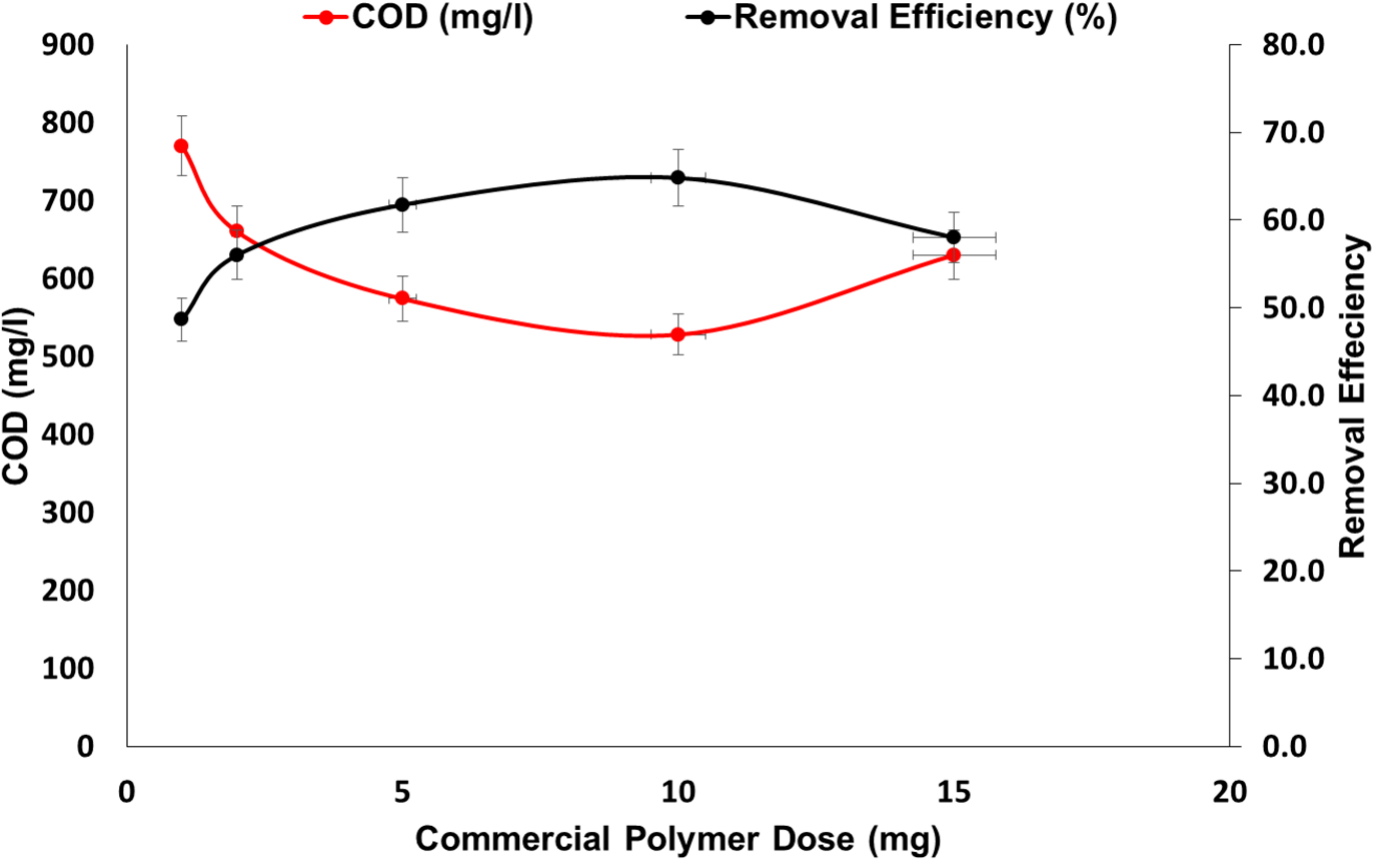
Effect of cationic surfactant CTAB dose integrated with alum on COD removal (pH 5.8, Initial COD 1500 mg/l, 800 mg Alum).

Table 3 and Fig. 15 show that the best alum dose (800 mg/L) was used in combination with a commercial cationic surfactant (CTAB) to improve the efficiency of removing food industry wastewater during the treatment process. The commercial cationic surfactant (CTAB) concentration ranges from 1 to 15 mg/L. COD concentrations were reduced from 1500 mg/L to 770, 660, 574, 523, and 630 mg/l using concentrations of commercial cationic surfactant (CTAB) of 1, 2, 5, 10 and 15 mg/L respectively. These results show that the best results for the removal of hazardous materials from food industry wastewater were obtained using alum (800 mg/L) integrated with CTAB at a concentration of 10 mg/L. The produced pH was around 5.8 (same result) at all levels of the concentrations used and this was the same observation after analysis of TDS that was produced from the treatment process, as shown in Table 2. The reduction of the turbidity was very significant as it was reduced by 95.4% after using 10 mg/L CTAB, however, the reduction was only 84.2% after using only 1 mg/L of CTAB.

**Table 3:** Treatment of food industry wastewater using optimum Alum (800 mg/L) with cationic flocculant CTAB.

Parameter	Raw wastewater	Flocculant dose (mg/L)				
		1	2	5	10	15
pH	6.7 ± 1.0	5.8 ± 0.9	5.7 ± 0.8	5.8 ± 0.9	5.7 ± 0.9	5.8 ± 1.1
NTU	266 ± 12	42 ± 4	29 ± 4	25 ± 6	12.1 ± 2	31 ± 8
TDS	770 ± 15	1130 ± 22	1200 ± 28	1190 ± 26	1210 ± 28	1220 ± 28
TSS	516 ± 24	280 ± 12	255 ± 14	220 ± 10	210 ± 16	260 ± 18
COD	1500 ± 124	770 ± 85	660 ± 66	574 ± 55	523 ± 52	630 ± 64

#### Coagulation-flocculation process using alum with bio-based cationic flocculant (BOMC)

**Figure 16:**
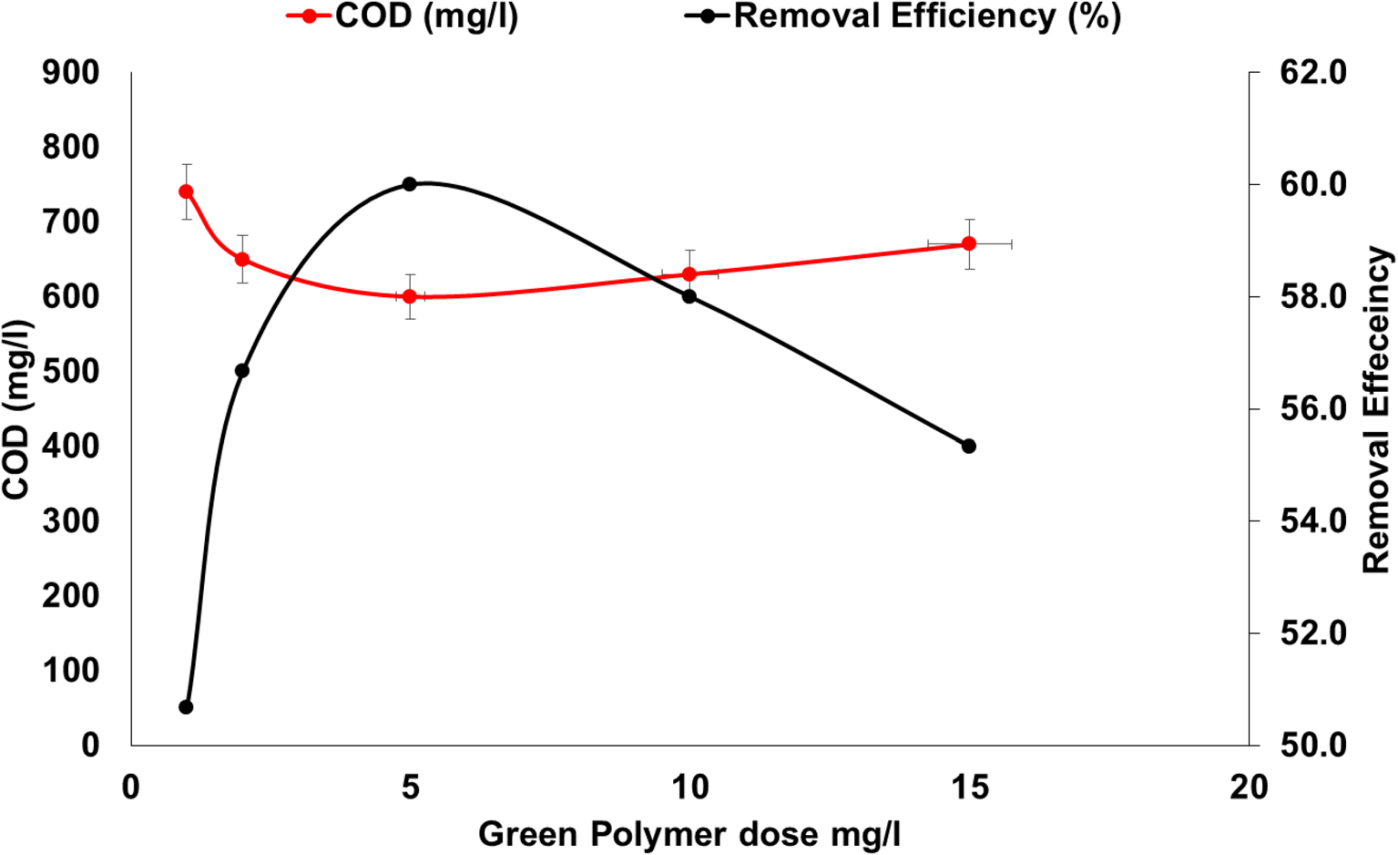
Effect of novel cationic surfactant BOMC dose integrated with alum on COD removal (pH 5.8, Initial COD 1500 mg/L, Alum 800 mg).

As shown in Fig. 16 and Table 4, the optimum alum dose (800 mg/L) was used integrated with green cationic flocculant BOMC to enhance the removal efficiency and the treatment process of food industry wastewater. The novel bio-based cationic flocculant (BOMC) concentration ranges from 1 to 15 mg/L. COD concentrations were reduced from 1500 mg/L to 740, 650, 580, 630, and 670 mg/L using concentrations of BOMC of 1, 2, 5, 10 and 15 mg/L respectively. These results show that the best results for the removal of hazardous materials from food industry wastewater were obtained using alum (800 mg/L) integrated with bio-based cationic surfactant BOMC with a concentration of 5 mg/L. The pH produced was around 5.8 (same result) at all levels of the concentrations used and this was the same observation after analysis of TDS that was produced from the treatment process as shown in Table 3. The reduction of the turbidity was very significant as it was reduced by 80% after using 5 mg/L of cationic surfactant, however, the reduction was only 72.9% after using only 1 mg/L of cationic flocculant. No studies have been found using these novel natural green materials as a flocculant, however, Shak and Wu^37^ treated palm oil mill effluent by using alum and *cassia obtusifolia* seed as a unique natural flocculant. It was found that using 1.15 g/L alum and 2.47 g/L *cassia obtusifolia* resulted in only 48.2% COD removal. However, in the current study, only 0.6 g/L alum was used. Kumar et al.^38^ investigated the use of *cassava peels* as a natural flocculant in combination with alum for treating institutional wastewater. The removal efficiency of COD reached 56.89% in optimum conditions, however, the removal efficiency of organic matter for the current study reached 61.3%.

**Table 4:** Treatment of food industry wastewater using optimum Alum (800 mg/L) with bio-based cationic flocculant BOMC.

Parameter	Raw wastewater	Novel flocculant dose (mg/L)				
		1	2	5	10	15
pH	6.7 ± 1.0	5.8 ± 1.1	5.7 ± 0.8	5.8 ± 0.9	5.7 ± 0.9	5.8 ± 1.1
NTU	266 ± 12	72 ± 8	60 ± 6	53 ± 6	55 ± 8	67 ± 10
TDS	770 ± 15	813 ± 22	818 ± 22	821 ± 24	815 ± 20	828 ± 22
TSS	516 ± 24	290 ± 16	255 ± 18	215 ± 16	235 ± 22	260 ± 20
COD	1500 ± 124	740 ± 78	650 ± 66	580 ± 58	630 ± 64	670 ± 66

### Economic cost for using commercial cationic flocculant (CTAB) and bio-based cationic flocculant (BOMC) as a flocculant

Recently, a comparison between commercial and chemical preparations for wastewater treatment have used in a wide range^39^, however, the economic aspect for treating industrial wastewater is considered urgent for research as reported from many studies as well^40–43^. The manufacturing cost of the bio-based cationic flocculant (BOMC) is evaluated and compared to the commercially manufactured flocculant (CTAB) to prove the extent of the benefit that can be obtained economically by achieving the same properties and using it in a practical application.

The cost of 1 g of the novel material according to the laboratory four synthesis steps was as follows. The first step to produce A.E.A, olive oil and E.D.A were used. The cost of the used quantity of olive oil was 0.02 $, while the cost of the used quantity from E.D.A was 0.03 $, so, to produce the required amount that used in the current study, the cost of A.E.A was 0.05 $. In the second step, the material resulting from the first step is added to cloro-acetyle chloride that cost 0.15 $, and following added to Dichloro-methane that cost 0.1 $, so, to produce C.A.E.O in the current study, it cost 0.3 $. In the third step, the material resulting from the second step is added to Morpheline that cost 0.05 $ in the current study, and this combination added to Ethylacetate that cost 0.03 in the current study, to produce M.A.E.O it cost 0.37 $. The certain amount of material resulting from the third step is added to Benzylchloride that cost 0.02 $ in the current study, and all combination added to acetnitril that cost 0.1 $ in the current study. So, the final cost of preparation 1 g of novel bio-based material is 0.49 $/gram, however, the commercial cationic flocculant cost 0.93 $ in the market in Egypt as it is proves the economically approach of the prepared material. Therefore, the bio-based cationic flocculant (BOMC) was cost less than the value of the manufacturing cost of the material used commercially. Moreover, using the novel material to treat wastewater have a dual benefits that it considering treating waste by waste to produce a clean water that it is considered sustainable benefits for environment and human.

## Conclusions

This study illustrates the effectiveness of a newly developed bio-based cationic flocculant, BOMC, in treating wastewater generated by the food industry. The study compares the performance of BOMC with that of the commercial cationic flocculant CTAB, when used in conjunction with alum. The results indicate a successful improvement in the flocculation process of food industry wastewater by utilizing alum and the new bio-based cationic flocculant. The optimal conditions for flocculation were found to be at pH 5.8, using a dose of 800 mg/L of alum and 10 mg/L of a commercial cationic flocculant, as compared to only 5 mg/L of a bioactive cationic flocculant that was prepared from COPO. Moreover, the economic cost of the new cationic flocculant was 0.49 $/g, which is much lower than the cost of the commercial cationic flocculant available in the market, which is 0.93 $/ g. This makes bio-based cationic flocculants a better option regarding environmental sustainability and cost-effectiveness.

## Data Availability

The datasets used and/or analyzed during the current study are available from the corresponding author upon reasonable request.
